# Re: Endourologic strategies for a minimally invasive management of urinary tract stones in patients with urinary diversion

**DOI:** 10.1590/S1677-5538.IBJU.2018.0123

**Published:** 2018

**Authors:** Ines Mendes Pina, Michael S. Floyd, Simon R. Stubington

**Affiliations:** 1 Department of Reconstructive Urology, St Helens & Knowsley Hospital NHS Trust, United Kingdom, UK; 2 Department of Urology, Michael Heal Unit, Mid Cheshire Hospital NHS Foundation Trust, United Kingdom, UK

*To the editor,*

We read with interest the recent paper by Zhong et al. examining the variety of endourological strategies available for the management of urinary tract stone disease in patients following urinary diversion (1).

The authors report a retrospective study involving 26 patients who underwent urinary diversion and who subsequently presented with stone related problems and allude to the variety of techniques available (1).

Specific to the lower tract it is stated that 3 patients underwent orthotopic neobladder surgery and were subsequently treated for vesical calculi with neobladder lithotripsy and in select cases a second look procedure was performed 3-5 days later (1).

The authors list the treatment modalities mentioned: PCNL, SWL, Percutaneous antegrade and retrograde ureteroscopy and open removal (1) but should acknowledge that in the diverted patient a variety of hybrid techniques have evolved to permit safe lower tract stone removal. Specific to the neuropathic patient with an ablated urethra and Mitrofanoff bladder laser cystolithotripsy with a flexible cystoscope (Leighton Technique) allowing complete stone removal in one sitting has been described (2). In the paediatric patient with an augmented bladder a separate hybrid technique involving endoscopic and laparoscopic approaches with preoperative lithotripsy has also been described (3). In patients with stone disease in a continent diversion another hybrid technique involving laparoscopic entrapment and fragmentation with conventional lithotripsy has been documented (4). The Mini PCNL technique has also been adapted for use in a spinal patient with an ablated urethra to achieve stone removal via a Mitrofanoff tract (5).
